# Enhancing the Cooling Efficiency of Aluminum-Filled Epoxy Resin Rapid Tool by Changing Inner Surface Roughness of Cooling Channels

**DOI:** 10.3390/polym16070874

**Published:** 2024-03-22

**Authors:** Chil-Chyuan Kuo, Hong-Wei Chen, Geng-Feng Lin, Song-Hua Huang, Shih-Feng Tseng

**Affiliations:** 1 Department of Mechanical Engineering, Ming Chi University of Technology, New Taipei City 24301, Taiwan; 2 Research Center for Intelligent Medical Devices, Ming Chi University of Technology, New Taipei City 24301, Taiwan; 3 Department of Mechanical Engineering, Chang Gung University, Taoyuan City 33302, Taiwan; 4 Center for Reliability Engineering, Ming Chi University of Technology, New Taipei City 24301, Taiwan; 5 Li-Yin Technology Co., Ltd., New Taipei City 24301, Taiwan; 6 Department of Mechanical Engineering, National Taipei University of Technology, Taipei City 106344, Taiwan

**Keywords:** surface roughness, cooling efficiency, aluminum-filled epoxy resin, rapid tool, cooling time, low-pressure wax injection molding

## Abstract

In low-pressure wax injection molding, cooling time refers to the period during which the molten plastic inside the mold solidifies and cools down to a temperature where it can be safely ejected without deformation. However, cooling efficiency for the mass production of injection-molded wax patterns is crucial. This work aims to investigate the impact of varying surface roughness on the inner walls of the cooling channel on the cooling efficiency of an aluminum-filled epoxy resin rapid tool. It was found that the cooling time for the injection-molded products can be determined by the surface roughness according to the proposed prediction equation. Employing fiber laser processing on high-speed steel rods allows for the creation of microstructures with different surface roughness levels. Results demonstrate a clear link between the surface roughness of cooling channel walls and cooling time for molded wax patterns. Employing an aluminum-filled epoxy resin rapid tool with a surface roughness of 4.9 µm for low-pressure wax injection molding can save time, with a cooling efficiency improvement of approximately 34%. Utilizing an aluminum-filled epoxy resin rapid tool with a surface roughness of 4.9 µm on the inner walls of the cooling channel can save the cooling time by up to approximately 60%. These findings underscore the significant role of cooling channel surface roughness in optimizing injection molding processes for enhanced efficiency.

## 1. Introduction

The cooling phase accounts for a significant portion of processing time in injection molding operations. In recent years, the adoption of conformal cooling channels (CCs) [1] in injection molds or dies has emerged as a widely recommended standard for enhancing mold capabilities. CC denotes a specialized type of cooling system tailored for dies or molds employed in diverse manufacturing processes, including blow molding [2], metal forming [3], plastic injection molding [4], die casting [5], and metal injection molding. The distinctive characteristic of CC lies in its capacity to enhance heat dissipation during the cooling phase across various manufacturing operations. CC is renowned for its capability to adapt to the shape of manufactured molded products.

Piekło et al. [6] analyzed the phenomenon of plasticity loss of steel core with CC. Results showed that the proposed methodology can determine the value of allowable deformations in the cooling channel zone. In a study by Nguyen et al. [7], CC was designed to enhance temperature distribution on the mold cavity surface during the injection molding process, demonstrating that the average temperature of the mold cavity surpassed that achieved with a conventional straight cooling channel. Vargas-Isaza et al. [8] assessed the cooling efficiency of polymer injection molds employing CC via numerical simulation. The results indicated a 9.26% reduction in warpage for the cup-shaped injection-molded part when CC was used, as opposed to the conventional cooling channel. Minh et al. [9] optimized injection mold cooling channels using Taguchi-integrated principal component analysis, revealing that CC resulted in an average temperature peaking at 58.78 °C. In the biomimetic engineering approach by Choi et al. [10], CC design led to a remarkable ten-fold reduction in pressure loss and approximately 46% improvement in temperature deviation compared to conventional cooling channels. Torres-Alba et al. [11] proposed an innovative CC system with high resistance to warping, demonstrating a 66% reduction in cycle time and an 81.88% decrease in residual stress. Gotlih et al. [12] introduced a CC system selection method based on non-dominated sorting, with simulation results indicating that it offers the lowest warpage and shortest cycle times. Kanbur et al. [13] focused on a plastic injection mold insert with CC, achieving deviations between printed and design parameters of less than 5% for circular and tapered channels in metal additive manufacturing. Torres-Alba et al. [14] introduced a new CC, reducing the cycle time of the injection-molded plastic part by about 32%. Torres-Alba et al. [15] presented a hybrid cooling model combining CC with mold inserts, enhancing the temperature map gradient and uniformity by approximately 51.666%. 

CC [16,17,18,19,20,21] is extensively employed in the injection molding because it provides cooling during the cooling stage. However, there is a limit to the cooling efficiency of CC. Therefore, developing technology for enhancing cooling efficiency is an important research issue if the mold industry officially wants to use aluminum-filled epoxy resin rapid tools for mass production. As the cooling water path is added with a characteristic structure [22], it can increase the contact area [23] for the coolant to take away the injection-molded product. This is expected to improve the cooling efficiency during the cooling stage after injection molding. In this study, different characteristic structures were created on the surface of the CC to change its surface roughness by a fiber laser patterning system [24]. Surface topography of the cooling channel was evaluated by laser scanning confocal microscope [25]. Effects of surface roughness of the inner wall of cooling channel on the cooling efficiency of aluminum-filled epoxy resin rapid tool [26] were investigated. 

## 2. Experiment

**Figure 1:**
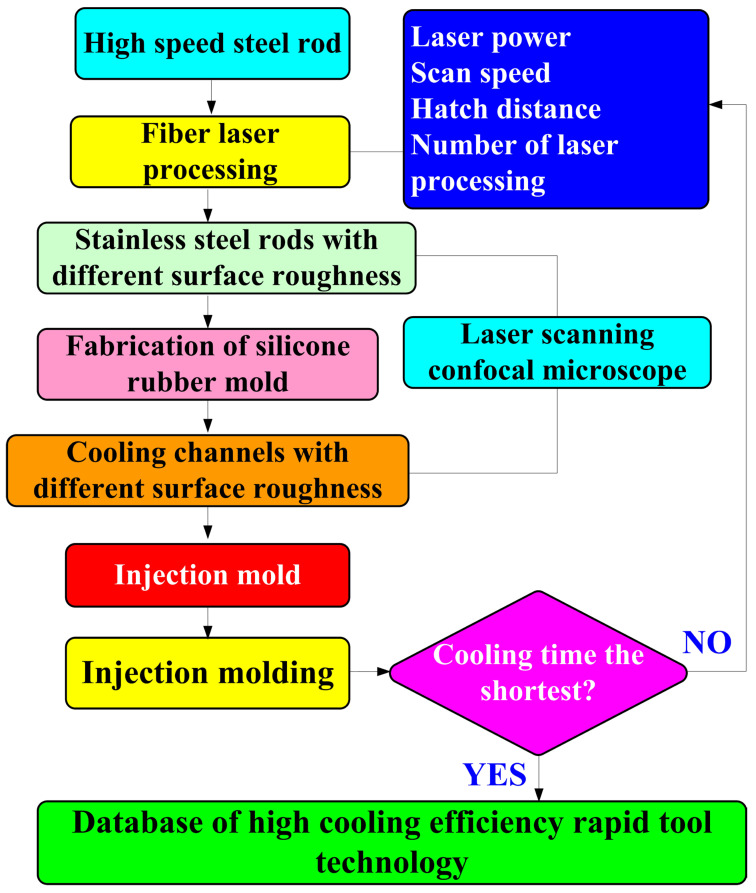
Flowchart of experimental methodology.

**Figure 2:**
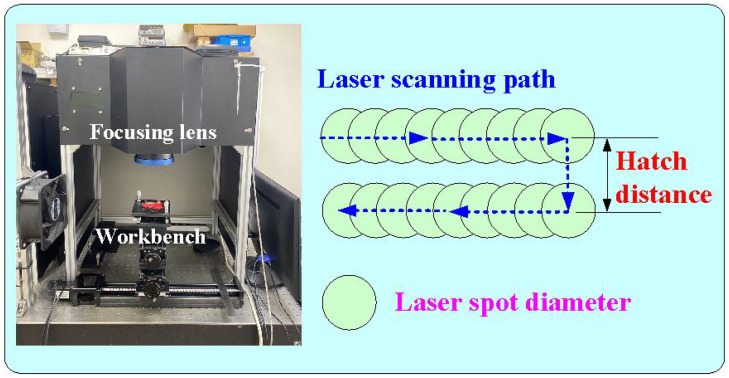
Processing paths for machining microstructures on the high-speed steel rod.

Figure 1 shows the flowchart of the experimental methodology. To create a characteristic structure on the surface of the CC and change its surface roughness, this study used a fiber laser patterning system (YLPN-1-4×200-30-M, IPG photonics Co., Ltd., Oxford, MA, USA) to pattern microstructures on the high-speed steel rods with a diameter and length of 10 mm and 100 mm, respectively. The focal length of the focus lens, the scanning field, the maximum scanning speed, and the spot diameter at the focusing point were 125 mm, 50 × 50 mm^2^, 6000 mm/s, and 40 μm, respectively. The fiber laser’s wavelength, maximum average power, maximum pulse repetition rate, and pulse duration were 1064 nm, 30 W, 1 MHz, and 4 ns, respectively. The specification of the fiber laser processing system and laser processing parameters are described in Table 1. A laser scanning confocal microscope (VK-X3000, Keyence Co., Ltd., Osaka, Japan) was employed to measure the surface roughness of the specimens and cooling channels. Surface average height, surface maximum height, surface peak-to-valley height, and surface developed area ratio of the specimens were analyzed. Optical microscope (Quick Vision 404, Mitutoyo Inc., Tokyo, Japan) and FE-SEM (JEC3000-FC, JEOL Inc., Tokyo, Japan) were used to investigate the morphology of the specimens and cooling channels. Figure 2 shows the processing path for machining microstructures in the high-speed steel rod. The laser power, scan speed, and hatch distance are 28 W, 30 mm/s, and 0.05 mm, respectively. The number of times laser processing is performed are 1, 3, 5, and 7 times.

**Figure 3:**
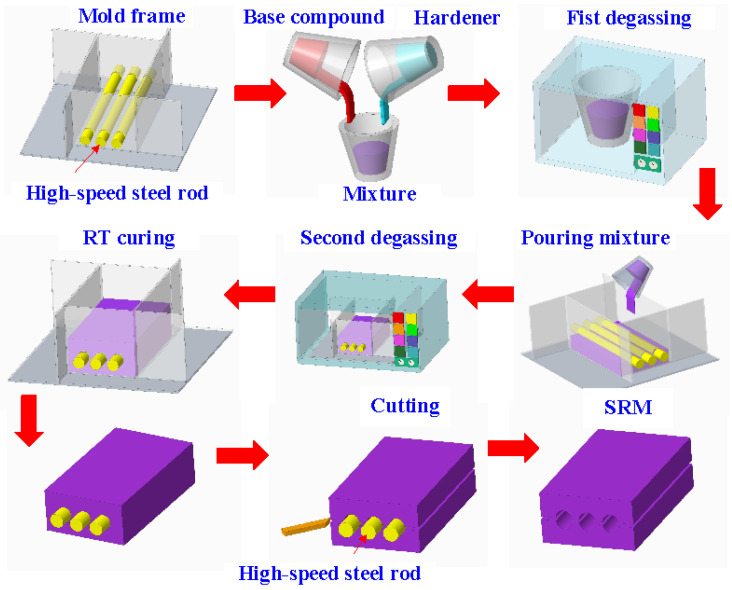
Manufacturing process of a silicone rubber mold for making wax cooling channel with microstructures.

**Figure 4:**
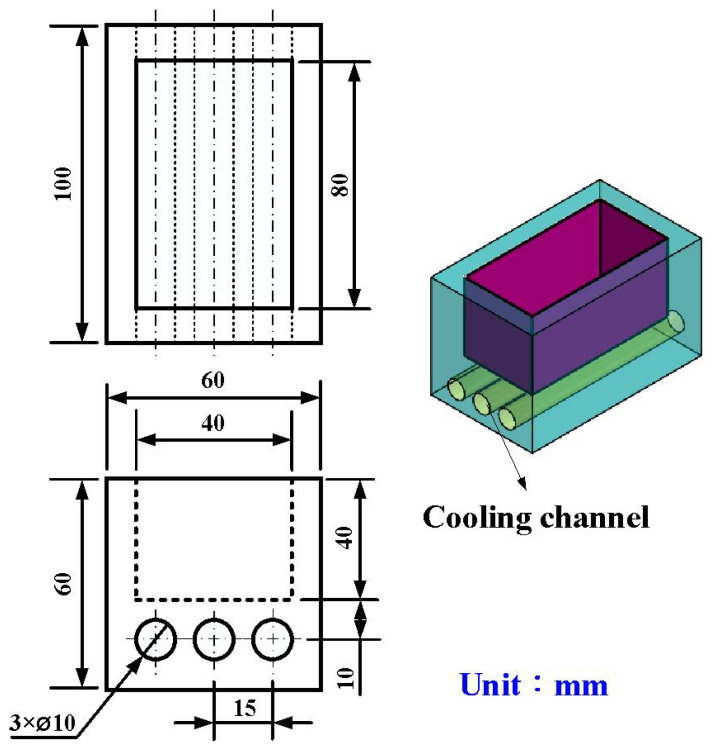
3D CAD model and dimensions of an aluminum-filled epoxy resin rapid tool with parallel CC.

**Figure 5:**
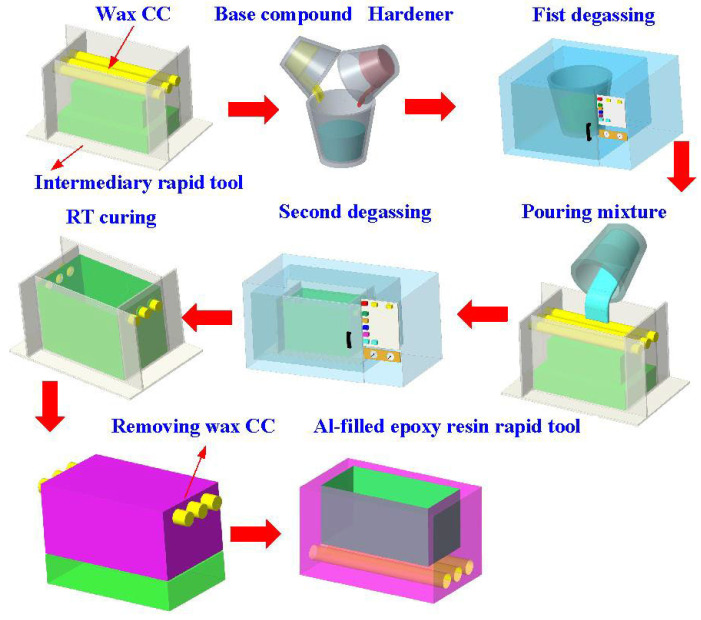
Manufacturing process of a rapid tool using an aluminum-filled epoxy resin.

**Figure 6:**
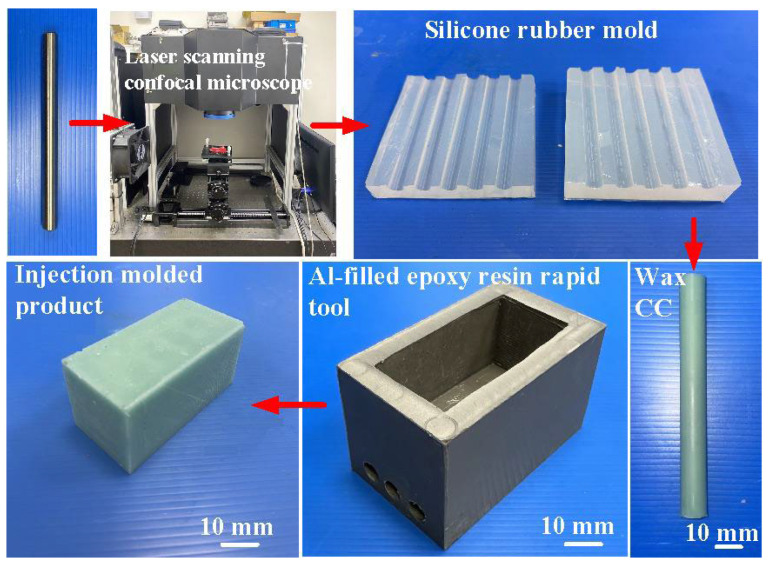
Research process of this study.

**Figure 7:**
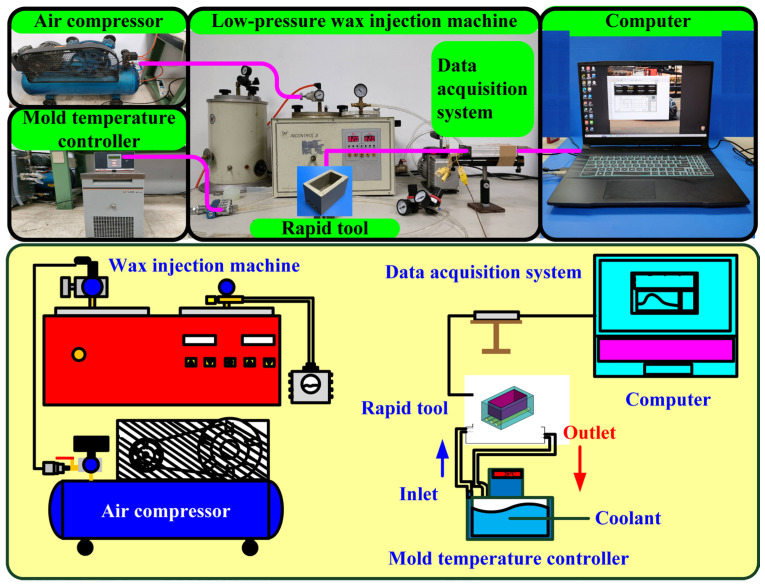
A homemade system for investigating the cooling time of the wax pattern after low-pressure wax injection molding.

Figure 3 shows the manufacturing process of a silicone rubber mold for making wax cooling channel with microstructures. The process of manufacturing silicone rubber molds typically comprises several sequential stages: (a) Master model preparation: This is initiated by readying the model or original object for replication by the mold. Such models can be designed from a range of materials like clay, wax, plastic, or even another silicone rubber mold. (b) Mold frame construction: A containment or frame, termed as a mold box, is erected around the model to encase the liquid silicone rubber during molding. Such boxes are commonly made from materials such as wood, plastic, or foam board. (c) Application of release agent: A substance known as a release agent is applied onto the model’s surface to prevent adhesion of the silicone rubber. Common agents include petroleum jelly, mold release sprays, or specialized silicone-based agents. (d) Silicone rubber mixing: Silicone rubber typically constitutes a two-part material comprising a base and curing agent, amalgamated in specific proportions as per the manufacturer’s instructions. Various formulations exist, such as tin-cure or platinum-cure, each possessing distinct curing characteristics. (e) Pouring silicone rubber: The amalgamated silicone rubber is poured into the mold box, completely covering the model. Care is taken to avoid entrapping air bubbles, which could lead to molding defects. (f) Curing: The silicone rubber undergoes curing or cross-linking, transitioning from a liquid to a solid rubber state. The duration of curing varies depending on factors like silicone rubber type, ambient temperature, and mold thickness. (g) Demolding: Following the complete curing of the silicone rubber, the mold is extracted from the mold box, and subsequently, the model is removed from the cured silicone mold. Demolding necessitates cautious handling to prevent harm to either the mold or the model, contingent upon the mold’s complexity and the silicone rubber’s flexibility. The length, width, and height of the silicone mold are approximately 100 mm, 100 mm, and 30 mm, respectively. The silicone rubber (KE-1310ST, Shin Etsu Inc., New Taipei City, Taiwan) and curing agent (CAT-1310S, Shin Etsu Inc., New Taipei City, Taiwan) were mixed in a weight ratio of 10:1 to manufacture a silicone rubber mold (SRM). The mixing process was conducted via a vacuum machine (F-600, Feiling, Inc., New Taipei City, Taiwan) to remove air bubbles derived from the mixing process. The wax (K512, Kato Inc., New Taipei City, Taiwan) was used as a molding material because the molded patterns can be employed for investment casting. Figure 4 shows the 3D CAD model and dimensions of an aluminum-filled epoxy resin rapid tool with parallel CC. There are three parallel CCs for cooling injection-molded products. The diameter of the CCs is about 10 mm. The length, width, and height of the mold cavity are approximately 80 mm, 40 mm, and 40 mm, respectively. The length, width, and height of the silicone mold are approximately 100 mm, 100 mm, and 60 mm, respectively. Figure 5 shows the manufacturing process of a rapid tool using an aluminum-filled epoxy resin containing about 70% aluminum powder by weight. The average particle size of aluminum powder is about 48 µm with a purity of about 96–99%. The curing agent (EP-2N-B, Ruixin Inc., Taipei, Taiwan) and base compound of the epoxy resin (EP-2N-A, Ruixin Inc., Taipei, Taiwan) were mixed to prepare matrix materials. The curing agent and epoxy resin were mixed in a weight ratio of 1:2. The mixture was stirred manually about 10 min until the mixture is well blended. Figure 6 shows the research process of this study. The high-speed steel rods were processed by a fiber laser machine to produce a master model with different surface roughness. A silicone rubber mold was then used to transfer the surface roughness of the steel rod to produce wax CCs with different surface roughness. Finally, an aluminum-filled epoxy resin rapid tool with CCs was fabricated after removing the wax CCs for low-pressure wax injection molding. A low-pressure wax injection molding process was used to assess the cooling performance of the fabricated aluminum-filled epoxy resin rapid tool. The horizontally oriented rapid tool received molten wax at 82 °C into a mold cavity set at 27 °C. Figure 7 shows a homemade system for investigating the cooling time of the wax pattern after injection molding via a low-pressure wax injection molding machine (0660, W&W Inc., New Taipei City, Taiwan). The aluminum-filled epoxy resin rapid tool is placed horizontally on the self-made platform. The low-pressure wax injection molding can be performed after the gate of the aluminum-filled epoxy resin rapid tool is aligned with the nozzle of the low-pressure wax injection molding machine. This homemade system involved a mold temperature controller (JCM-33A, Shinko Inc., New Taipei City, Taiwan), a K-type thermocouple (C071009-079, Cheng Tay Inc., New Taipei City, Taiwan) with a measurement sensitivity of ±1 °C and a coolant reservoir with a thermo-electric cooler (TEC12706AJ, Caijia Inc., Taipei City, Taiwan). The temperature history was recorded using a data acquisition system (MRD-8002L, IDEA System Inc., New Taipei City, Taiwan). The coolant flow rate is about 4 L/min. The wax injection pressure is approximately 0.06 MPa. The ejection temperature for the molded wax pattern was set at 30 °C through a series of test runs. The inlet coolant temperature was kept at 27 °C. The temperature histories of the molded wax patterns were recorded using temperature sensors. The ambient temperature was maintained at 27 °C.

## 3. Results and Discussion

**Figure 8:**
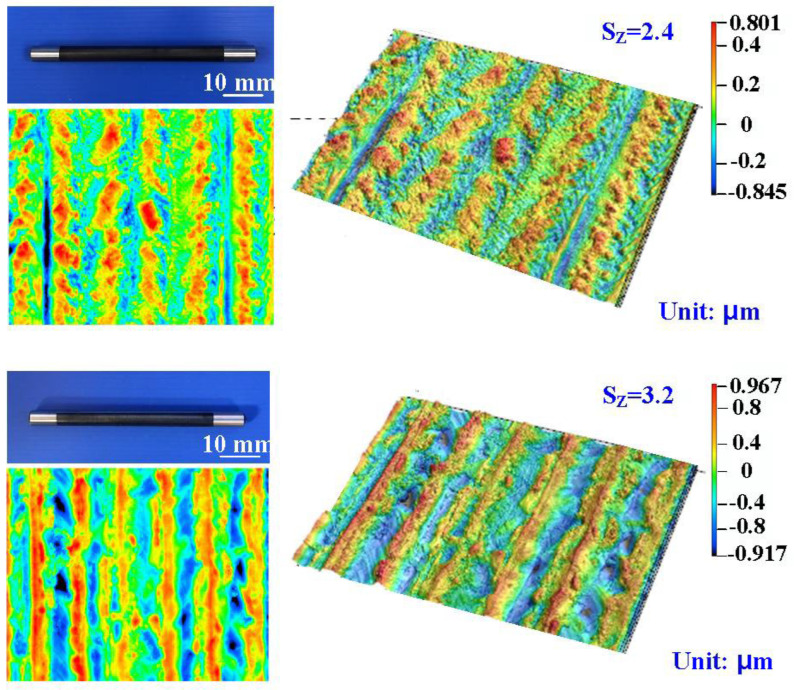

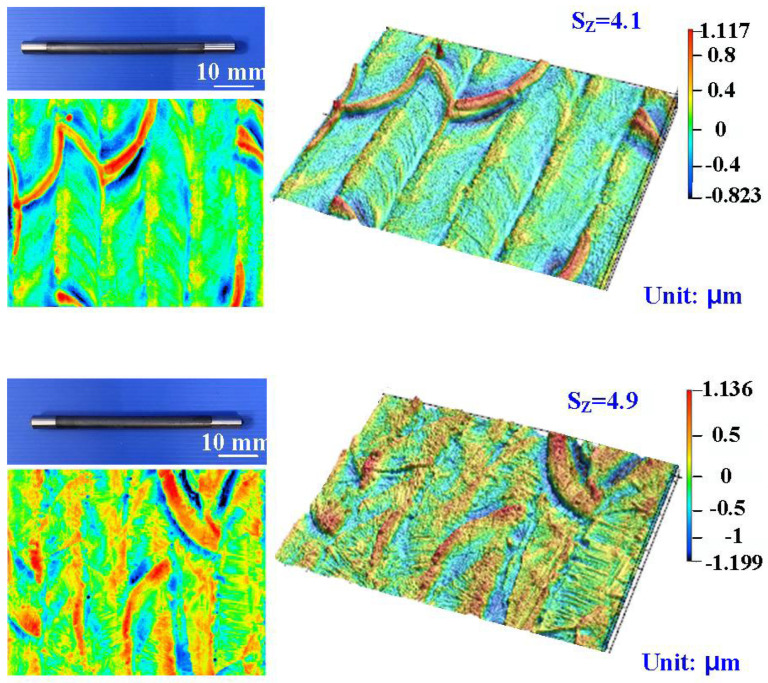
Surface roughness of the high-speed steel rods after fiber laser processing.

Figure 8 shows the surface roughness of the high-speed steel rods after fiber laser processing. The image shows the 3D presentation of the high-speed steel rods. The numbers assigned to laser processing cycles are 1, 3, 5, and 7, respectively. The Sz surface roughness are about 2.4 μm, 3.2 μm, 4.1 μm, and 4.9 μm, respectively. The Sz stands for the maximum height and is defined as the sum of the most significant peak height value and the most considerable pit depth value within the measurement area. 

**Figure 9:**
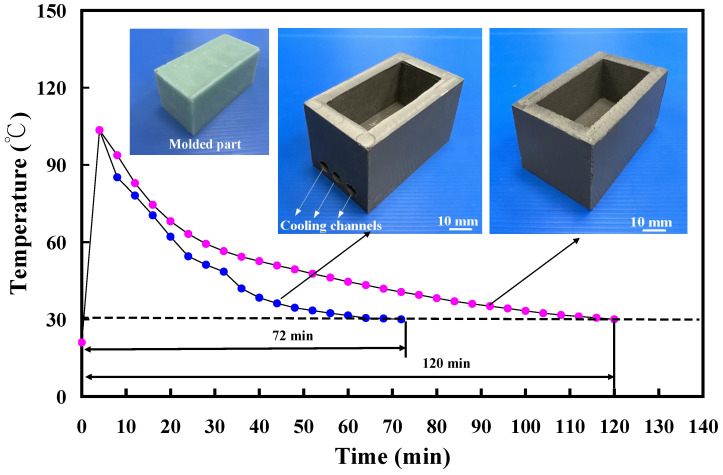
Cooling time of the molded wax pattern using aluminum-filled epoxy resin rapid tool with and without cooling channels.

**Figure 10:**
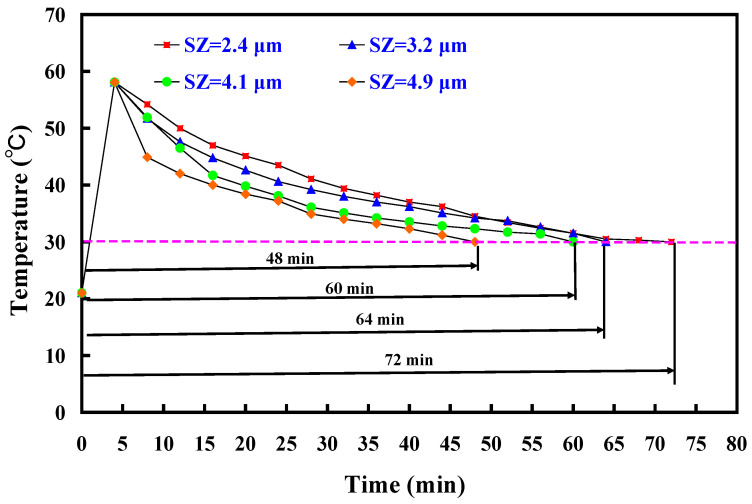
Cooling time of the molded wax pattern using different surface roughness of the inner wall of the cooling channel inside the aluminum-filled epoxy resin rapid tool.

Figure 9 depicts the cooling time comparison of molded wax patterns when utilizing an aluminum-filled epoxy resin rapid tool with and without cooling channels. The findings highlighted that the presence of cooling channels in the aluminum-filled epoxy resin rapid tool significantly reduces the cooling time for injection-molded products. Specifically, the cooling time for injection-molded products is approximately 120 min when employing an aluminum-filled epoxy resin rapid tool without cooling channels in low-pressure injection molding. In contrast, the cooling time decreases to approximately 72 min when using a rapid tool equipped with cooling channels, demonstrating a substantial 40% reduction in the cooling stage for injection-molded products. Figure 10 illustrates the cooling time variation in molded wax patterns when employing an aluminum-filled epoxy resin rapid tool with different Sz surface roughness on the inner wall of the cooling channel. The findings demonstrated that the cooling time for the injection-molded product varies, measuring approximately 72 min, 64 min, 60 min, and 48 min when utilizing a tool with maximum heights of 2.4 μm, 3.2 μm, 4.1 μm, and 4.9 μm on the inner wall of the cooling channel. Three noteworthy phenomena are revealed. Firstly, the surface roughness of the inner wall of the cooling channel directly influences the cooling time of the injection-molded product. This suggests that higher surface roughness facilitates faster heat transfer, leading to more efficient cooling and consequently shorter production cycles. Secondly, as the maximum height of the cooling channel increases, the cooling time for the injection-molded product decreases. Specifically, utilizing an aluminum-filled epoxy resin rapid tool with a maximum height of 4.9 μm results in a cooling time saving of about 34% compared to a tool with a maximum height of 2.4 μm. Thirdly, when comparing a tool with a maximum height of 4.9 μm to a tool without cooling channels for low-pressure injection molding, a cooling time saving of up to 60% is achieved in the cooling stage. Therefore, the effect of surface roughness on cooling efficiency is meticulously detailed by observing variations in cooling time among molded wax patterns. The investigation demonstrated that different surface roughness values on the inner wall of the cooling channel exert a direct influence on the cooling time of the injection-molded product. Specifically, it is revealed that as the maximum height of the Sz surface roughness increases, the cooling time decreases. This correlation suggests that higher surface roughness facilitates faster heat transfer, thereby enhancing cooling efficiency and consequently shortening production cycles. Furthermore, this work quantifies the cooling time savings associated with increasing surface roughness values, thereby highlighting the practical implications of the findings. Through the comparative analyses of different surface roughness values, the significant impact of surface roughness on cooling efficiency in the context of low-pressure wax injection molding processes is effectively illustrated. Overall, these results emphasize the importance of surface roughness [27,28,29] as a crucial factor in optimizing cooling efficiency and reducing production time in manufacturing applications. These results are also supported by the results from Yang et al. [30] due to rotating flow influencing the overall dynamics of the coolant.

**Figure 11:**
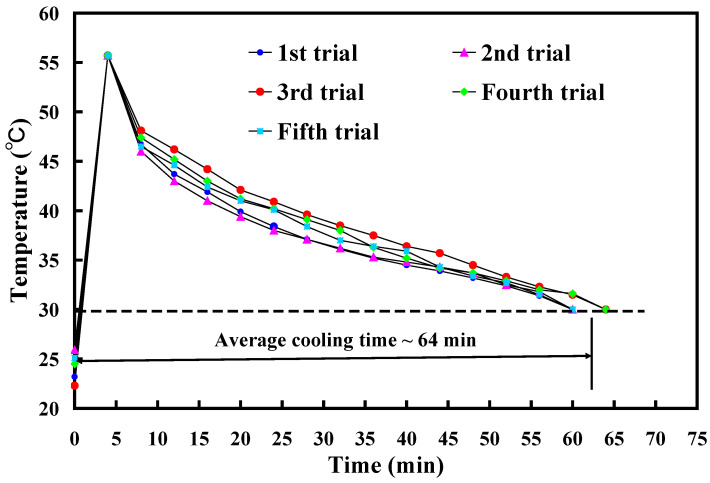
Results of injection-molded product cooling time reproducibility test.

**Figure 12:**
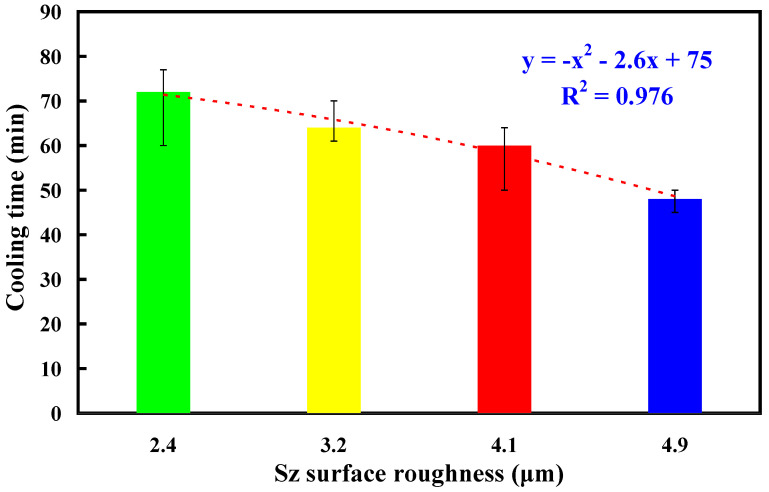
Experimental results of cooling time reproducibility of injection-molded products.

To assess the reproducibility of cooling times for injection-molded products, this study used an aluminum-filled epoxy resin rapid tool with Sz surface roughness of 3.2 μm to conduct five repetitions of low-pressure wax injection molding. Figure 11 shows the results of injection-molded product cooling time reproducibility test. Five trials were performed in this study. As can be seen, the average cooling time is about 64 min. This result shows that the cooling time of injection-molded products is repeatable. Figure 12 shows the experimental results of cooling time reproducibility of injection-molded products. The results showed that the average cooling times for the injection-molded products are approximately 72 min, 64 min, 60 min, and 48 min corresponding to the aluminum-filled epoxy resin rapid tool with Sz surface roughness of 2.4 μm, 3.2 μm, 4.1 μm, and 4.9 μm, respectively. It should be noted that the average cooling time for the injection-molded products (y) can be determined by the Sz surface roughness (x) according to the prediction equation of y = −x^2^ − 2.6x + 75 with a correlation coefficient of 0.976. 

**Figure 13:**
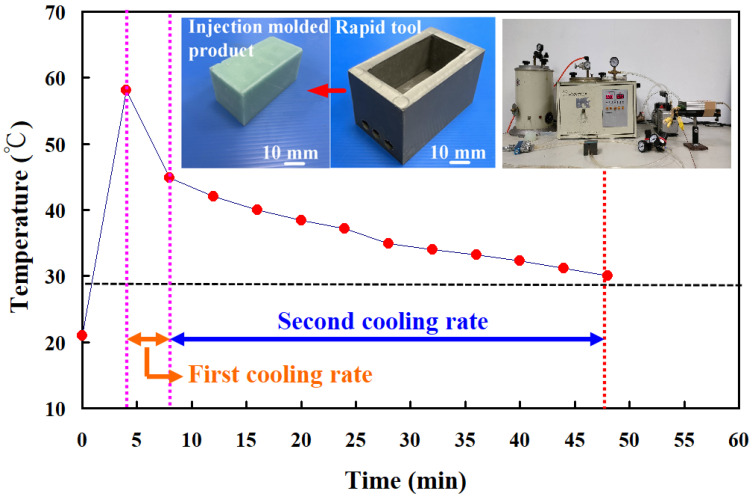
Two cooling rates in the cooling stage after low-pressure wax injection molding using an aluminum-filled epoxy resin rapid tool.

**Figure 14:**
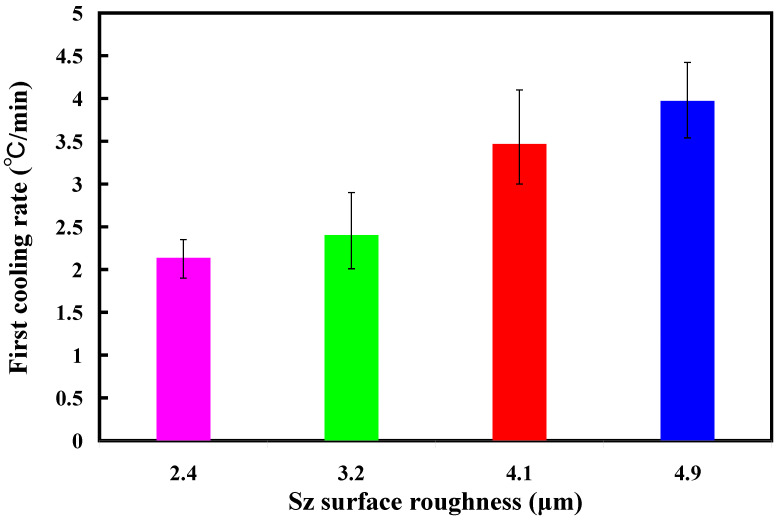
First cooling rate in the cooling stage after low-pressure wax injection molding using an aluminum-filled epoxy resin rapid tool.

**Figure 15:**
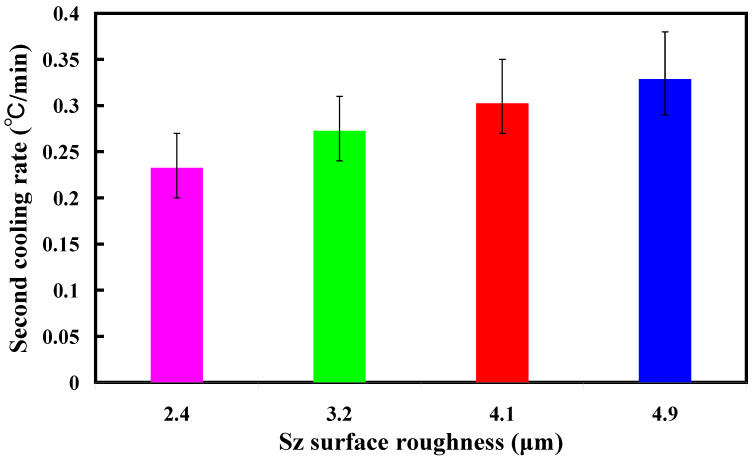
Second cooling rate in the cooling stage after low-pressure wax injection molding using an aluminum-filled epoxy resin rapid tool.

**Figure 16:**
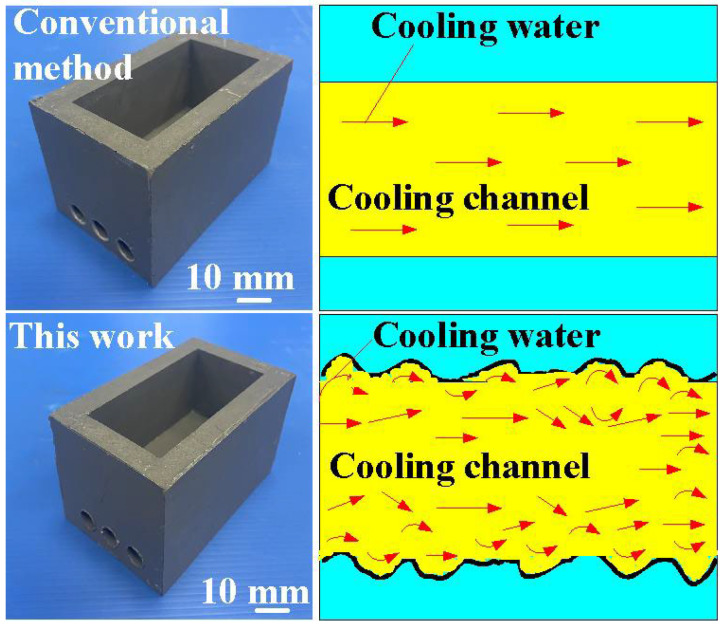
Cooling mechanisms for aluminum-filled epoxy resin rapid tool proposed by this work compared with conventional method.

Figure 13 shows the two cooling rates in the cooling stage after low-pressure wax injection molding using an aluminum-filled epoxy resin rapid tool. The first cooling rate is defined as eight minutes before the cooling time of the low-pressure injection-molded product. The relationship between the cooling temperature of the injection-molded product and the cooling time. The second cooling rate is defined as eight minutes after the cooling time of the low-pressure injection-molded product. The relationship between the cooling temperature of the injection-molded product and the cooling time. As can be seen, the first cooling rate is faster, and the second cooling rate is slower. Figure 14 shows the first cooling rate in the cooling stage after low-pressure wax injection molding using an aluminum-filled epoxy resin rapid tool. The results showed that the shorter the cooling time of the injection-molded product, the higher the first cooling rate. The results showed that the first cooling rate of the injection-molded product is approximately 2.13 °C/s, 2.396 °C/s, 3.462 °C/s, and 3.966 °C/s when the aluminum-filled epoxy resin rapid tool with Sz surface roughness of 2.4 μm, 3.2 μm, 4.1 μm, and 4.9 μm on the inner wall of the cooling channel are used for low-pressure injection molding, Figure 15 shows the second cooling rate in the cooling stage after low-pressure wax injection molding using an aluminum-filled epoxy resin rapid tool. The results showed that the shorter the cooling time of the injection-molded product, the higher the second cooling rate. The results showed that the second cooling rate of the injection-molded product is approximately 0.232 °C/s, 0.272 °C/s, 0.302 °C/s, and 0.328 °C/s when the aluminum-filled epoxy resin rapid tool with Sz surface roughness of 2.4 μm, 3.2 μm, 4.1 μm, and 4.9 μm on the inner wall of the cooling channel are used for low-pressure injection molding. Laminar flow refers to the smooth, orderly movement of a liquid in parallel layers. In laminar flow, the fluid moves in a well-organized and predictable manner, and this results in minimal turbulence across the flow direction. Turbulent flow [31] is a type of fluid flow characterized by chaotic, irregular movement of the fluid particles. In contrast to laminar flow, where fluid moves smoothly in parallel layers, turbulent flow is characterized by swirling and unpredictable motion. This chaotic behavior leads to the mixing of fluid particles and increased momentum exchange within the flow. Thus, turbulent flow enhances heat transfer compared to laminar flow, and this increased heat transfer is beneficial for more efficient cooling. Figure 16 illustrates the cooling mechanisms for the different surface roughness of the inner wall of cooling channel on the cooling efficiency of aluminum-filled epoxy resin rapid tool. The contact area for carrying away the cooling channel can be enhanced by increasing the feature structure of the cooling channel, improving the cooling efficiency.

According to the above research results, the findings of this work highlight the significant potential applications in the investment casting industry, mainly due to the notable impact of reduced cooling times on production costs during the mass production of wax patterns [32,33,34,35]. Wax patterns are versatile tools used in industries such as dentistry, jewelry making, and manufacturing for creating intricate prototypes and components with precision. Whether in dental prosthetics, jewelry design, or industrial casting, wax patterns serve as the foundation for shaping and refining intricate details before final production. In general, reducing the cooling time of the molded part is a crucial aspect of optimizing the injection molding process. Shorter cooling times can increase production efficiency and reduce cycle times, allowing for higher throughput in the manufacturing process. Based on green manufacturing [36,37,38], mechanical engineers or mold designers carefully consider these factors to determine the optimal cooling time for a specific injection molding application. Based on the aforementioned results, this study highlights its most significant practical implications within the mold or dies industry. In general, the mission of coolant in injection molding is to ensure precise temperature management throughout the process, leading to enhanced product quality, reduced cycle times, prolonged tool life, and improved overall efficiency in manufacturing. One of the sustainable development goals is environmental degradation. Hence, the findings of this research possess practical relevance for industry application and align with sustainable development goals 7, 9, 10, and 12 [39]. The research implemented water as the coolant. However, hydrogen [40,41,42] is favored for its superior thermal conductivity as a high-performance gaseous cooling medium in industrial application. Additionally, helium gas [43] finds extensive use in gas-cooled nuclear reactors due to its low neutron absorption tendencies. Sulfur hexafluoride is another prevalent choice for cooling various high-voltage power systems like circuit breakers, transformers, or switches [44,45]. Ongoing investigations into these matters will be presented in a subsequent study.

## 4. Conclusions

In the context of low-pressure wax injection molding, cooling time pertains to the duration in which the molten plastic within the mold undergoes solidification and cools to a temperature that allows safe ejection without deformation. The main objective of this study is to investigate the effects of surface roughness of the inner wall of cooling channel on the cooling efficiency of aluminum-filled epoxy resin rapid tool. The main conclusions from the experimental work in this study are as follows:

1. The results of this study underscore the substantial potential applications within the investment casting industry, particularly attributed to the noteworthy effects of decreased cooling times on production costs during the mass manufacturing of wax patterns.
2. The results showed that the application of fiber laser processing on the surface of high-speed steel rods allows for the development of microstructures with diverse surface roughness. The average cooling time for the injection-molded products (y) can be determined by the Sz surface roughness (x) according to the prediction equation of y = −x^2^ − 2.6x + 75 with a correlation coefficient of 0.976.
3. The surface roughness of the inner walls of the cooling channel significantly impacts the cooling duration of injection-molded items. In contrast to low-pressure wax injection molding using an aluminum-filled epoxy resin rapid tool with a surface roughness of 2.4 μm on the cooling channel’s inner walls, employing an aluminum-filled epoxy resin rapid tool with a surface roughness of 4.9 μm for low-pressure wax injection molding can result in time savings and an improvement in cooling efficiency of approximately 34%.
4. The utilization of an aluminum-filled epoxy resin rapid tool with a surface roughness of 4.9 μm on the inner walls of the cooling channel can reduce cooling time up to approximately 60% compared to low-pressure wax injection molding using an aluminum-filled epoxy resin rapid tool without a cooling channel.

## Figures and Tables

**Table 1:** Specification of the fiber laser processing system and laser processing parameters.

Parameter	Value
Spot diameter (μm)	40
Maximum average power (W)	30
Wavelength (nm)	1064
Pulse duration (ns)	4
Focal length (mm)	125
Maximum pulse repetition rate (MHz)	1
Processing laser power (W)	28
Hatch distance (mm)	0.05
Number of laser processing	1, 3, 5, 7

## Data Availability

Data are contained within the article.
